# The effectiveness of intermittent theta burst stimulation for upper limb motor recovery after stroke: an exploratory randomized controlled trial

**DOI:** 10.3389/fneur.2025.1634277

**Published:** 2025-09-12

**Authors:** Songbin Chen, Xiaotong Li, Wenqing Yang, Guiyuan Cai, Shunxi Zhang, Yujie Chen, Wenyu Chen, Frank Kulwa, Huangjie Huang, Lanfang Xie, Lingling Tian, Yangkang Zeng, Hai Li

**Affiliations:** ^1^Neurorehabilitation Laboratory, Department of Rehabilitation Medicine, Shenzhen Hospital, Southern Medical University, Shenzhen, China; ^2^Department of Rehabilitation Medicine, Guangzhou First People’s Hospital, School of Medicine, South China University of Technology, Guangzhou, China; ^3^Clinical School of Acupuncture, Moxibustion and Rehabilitation, Guangzhou University of Chinese Medicine, Guangzhou, China; ^4^Department of Rehabilitation Medicine, The First Affiliated Hospital, Sun Yat-sen University, Guangzhou, China; ^5^Guangdong Provincial Key Lab of Robotics and Intelligent System, Shenzhen Institutes of Advanced Technology, Chinese Academy of Sciences, Shenzhen, China

**Keywords:** iTBS, stroke, motor function, muscle tone, functional connectivity

## Abstract

**Background:**

Stroke often results in significant motor impairments, particularly in the upper limbs, which can severely impact functional independence and quality of life. Conventional rehabilitation methods provide limited recovery, necessitating the exploration of adjunctive therapies to enhance motor function. Intermittent theta burst stimulation (iTBS) is a brain stimulation technique that has shown promise in improving motor function after stroke. This study was conducted to investigate whether iTBS targeting ipsilesional primary motor cortex can induce improvements of the paretic upper limb and physiological changes in cortical excitability in subacute stroke patients.

**Methods:**

50 patients were randomized assigned to either iTBS or sham stimulation across 10 sessions. Motor function, symptom severity, muscle tone, and functional independence were evaluated. Additional measures included rest motor threshold (RMT), oxygenated hemoglobin concentration.

**Results:**

Both the iTBS and sham groups showed significant improvements in National Institutes of Health Stroke Scale (NIHSS) (iTBS: *p* = 0.002; sham: *p* = 0.039), Fugl-Meyer Assessment (FMA) (iTBS: *p* < 0.001; sham: *p* = 0.005), and Modified Barthel Index (MBI) (iTBS: *p* < 0.001; sham: *p* = 0.002) scores post-intervention. Only the iTBS group demonstrated significant improvements in Modified Ashworth Scale (MAS) (*p* < 0.001), Wolf Motor Function Test (WMFT) (*p* < 0.001), and RMT (*p* = 0.016). The iTBS group exhibited a trend toward greater improvements in MAS (*p* = 0.001), WMFT (*p* = 0.002), and MBI (*p* < 0.001). RMT in contralateral Primary motor cortex (M1) was significantly lower in the iTBS group (*p* = 0.016), and functional connectivity between each M1 regions was notably enhanced (*p* = 0.049).

**Conclusion:**

These findings suggest that iTBS may offer additional benefits in improving functional task performance and cortical connectivity in subacute stroke patients.

**Clinical trial registration:**

https://www.chictr.org.cn/showproj.html?proj=193454, Identifier ChiCTR2300072415.

## 1 Introduction

Stroke is the most prevalent and severe neurological condition globally, contributing significantly to the worldwide health burden (1). After 3–6 months onset of stroke, over half of ischemic stroke survivors continue to experience residual upper limb sensory and motor dysfunction, seriously impacting their quality of life (2). It has been shown that the balance of cortical excitability is altered in stroke patients, as evidenced by decreased excitability in the affected hemisphere and hyperexcitability in the unaffected hemisphere. Two main theoretical models are currently applied in the recovery of upper limb motor function after a stroke. The first is the interhemispheric rivalry model, which suggests that the intact hemisphere (IH) may inhibit the excitability of the stroke-affected hemisphere (SH) through transcallosal inhibition. Enhancing SH excitability by either stimulating the SH or inhibiting the IH may improve hand function. The second is the vicariation model, which proposes that increased IH excitability compensates for the loss in SH, aiding in functional recovery post-stroke (3, 4).

Repetitive transcranial magnetic stimulation (rTMS), a non-invasive brain stimulation technique, is increasingly reported as a promising intervention that improves motor performance of affected upper limb in stroke by modulating cortical excitability (5). Research has shown that limitations in the recovery of functional hand movements, despite intensive rehabilitation, are often linked to abnormal cortical activity patterns (6). Previous studies indicated that the excitability and connectivity of the primary motor cortex (M1) with remote areas decrease in the initial weeks after stroke but gradually increase as motor function improves (7). Several studies have shown that corticospinal excitability is lower in ipsilesional M1 (iM1) compared to contralesional M1 (cM1) (8). Intermittent theta burst stimulation (iTBS) is a variant of rTMS protocol, which requires a lower stimulation intensity in a shorter time to achieve similar therapeutic effect in stroke patients (9). As a safer and more effective stimulation, iTBS is currently being tested in pilot studies to improve limb motor function and activities of daily living (ADL) in chronic stroke patients (10, 11). It is suggested that integrating brain stimulation like iTBS with existing rehabilitation protocols, along with behavioral interventions, could enhance clinical outcomes by maximizing cortical plasticity.

Talelli et al. conducted a study on the effects of iTBS on hand function in chronic stroke patients, comparing it with sham stimulation (4). They found a decrease in reaction time and an increase in motor evoked potential (MEP) amplitude in the affected hand. However, the small sample size and lack of repeated treatments limit the potential for long-lasting changes. Watanabe et al. investigated the efficacy of SH motor cortex iTBS stimulation in acute ischemic stroke patients, along with IH 1 Hz rTMS stimulation. The iTBS group exhibited increased scores on finger function tests, whereas 1 Hz rTMS stimulation led to reduced Modified Ashworth Scale (MAS) scores for wrist and finger (12). Volz et al. investigated the effects of iTBS or sham stimulation administered to stroke patients within 2 weeks post-stroke, prior to conventional physical therapy, over 5 days. The real stimulation group showed significantly greater grip strength recovery and stronger connectivity in the iM1 of the affected hemisphere (13). Subacute phase is considered critical for neuroplasticity and spontaneous recovery, where interventions may yield the most significant functional gains. Compared to the chronic phase, the subacute stage offers a more dynamic environment for brain reorganization, and early neurorehabilitation has been shown to improve outcomes (2). However, there is still a lack of sufficient research and sample size to compare sessions of iTBS with sham stimulation in subacute stroke patients. It is still unclear how iTBS and occupational therapy specifically influence movements and corticospinal excitability in the subacute phase of upper limb paralysis rehabilitation. Our objective was to investigate whether iTBS can safely induce improvements in the motor behavior of the paretic upper limb and to identify the physiological changes in cortical excitability in stroke patients with subacute cerebral infarction.

## 2 Materials and methods

### 2.1 Participants

Fifty stroke patients with a first-ever ischemic stroke were recruited from Guangzhou First People’s Hospital. The inclusion criteria were as follows: (1) age ≥ 18; (2) ischemic stroke confirmed by DWI; (3) onset of stroke within 2 weeks to 6 months (14, 15); (4) unilateral upper limb motor impairment; (5) no severe aphasia, apraxia, and neglect; (6) signed informed consent. The exclusion criteria were: (1) contraindications for rTMS; (2) severe general impairment or serious medical conditions; (3) cranial bone defects. Informed consent was obtained from all participants, and the study was approved by the Guangzhou First People’s Hospital Human Research Ethics Committee, with approval number K-2023-021-02, adhering to the Declaration of Helsinki.

### 2.2 Study design

We conducted a single-center, prospective, randomized, sham controlled clinical trial with a double-blind design from January 2023 to July 2024. Eligible participants were recruited by an independent therapist and randomly allocated in a 1:1 ratio to either iTBS group or sham group. Randomization was performed using computer-generated sequences, and allocation concealment was ensured via sequentially numbered, opaque, sealed envelopes prepared by an independent researcher not involved in the intervention or assessment. Outcome measures were assessed at baseline (T1) and after the 10^th^ session (T2) by a senior occupational therapist blinded to the group assignment. Patients themselves were also unaware of their group assignment.

### 2.3 Intervention

iTBS was administered over the iM1 using a shape-8 coil with a Magnetic Stimulator (YIRUIDE Medical Co., Wuhan, China). The coil was oriented tangentially to the scalp, with the handle directed superiorly. The iTBS pattern comprises bursts containing three pulses at 50 Hz repeated at 5 Hz, and each session consisted of a total of 600 pulses delivered over approximately 3 min. A 2-s train of TBS was repeated every 10 s for a total duration of 192 s (9). The stimulation site on the iM1 was determined as the location producing the largest MEP amplitude in the paretic first dorsal interosseous (FDI) muscle, referred to as the “hotspot.” Rest motor threshold (RMT) was the most common measures of corticospinal excitability (3). Surface electromyography was recorded from the FDI of the paretic hand. Stroke survivors were seated in a comfortable chair with hand and back support (16). RMT was defined as the lowest stimulus intensity required to evoke a MEP > 50 μV in 5 out of 10 trials. When no MEPs could not be elicited in the paretic FDI, iTBS was applied to the iM1 at the mirror location of the “hotspot” for the cM1. The optimal position was marked on the scalp. The iTBS stimulation intensity was set to 70% RMT in the IH based on established safety and efficacy parameters reported in prior clinical studies (9, 17). For those individuals in whom MEPs could not be elicited in the SH even at 100% maximum stimulator output (MSO), RMT was considered 100% MSO. As 40% MSO is the upper limit for iTBS, the stimulation intensity was kept at 40% MSO if the calculated intensity exceeded this value. Sham iTBS as administered with the coil held at a 90° angle to the scalp, but used the same stimulation parameters and sounds as the real iTBS. After stimulation, all patients received standard rehabilitation training within 5 min, which consisted of 30-min sessions once daily, 5 days per week, for two consecutive weeks. The training included upper limb motor function exercises, task-specific functional training, and occupational therapy. This individualized rehabilitation minutes, was administered by an experienced therapist, who were unaware of the group assignment.

### 2.4 Assessment

**Figure 1:**
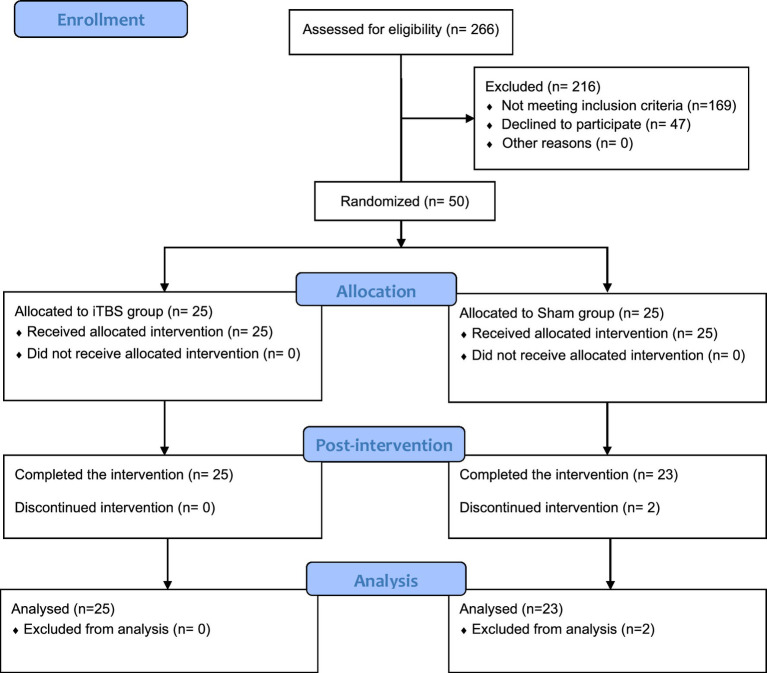
Consolidated standards of reporting trials flow diagram. iTBS, Intermittent theta burst stimulation.

Assessments were administered at T1 and T2 by three experienced raters, blinded raters. The degree of motor impairment in stroke patients was assessed by two experts. The primary outcome was the Fugl-Meyer Assessment (FMA-UE). The secondary outcomes included the National Institutes of Health Stroke Scale (NIHSS), Modified Ashworth Scale (MAS), Modified Barthel Index (MBI), Wolf Motor Function Test (WMFT), and RMT. The FMA-UE evaluates upper limb motor skills with a maximum score of 66 points (18). The NIHSS is a tool used to quantify the severity of neurological deficit post stroke. The Wolf Motor Function Test (WMFT) assesses upper extremity performance with a maximum score of 75 (19). The MAS rates muscle tone of wrist flexors from 0 (normal) to 4 (rigid) (20). The Modified Barthel Index (MBI), a total score of 100, assesses the self-care abilities of stroke patients by evaluating 10 ADL and mobility, rating the level of assistance required for each (21). Cortical excitability was assessed using functional near-infrared system (fNIRS) and RMT by W. Y. Chen and G. Y. Cai. The NirScan-6000A system (Danyang Huichuang Medical Equipment Co., Ltd., Jiangsu, China) was used to collect and record near-infrared data from subjects in a resting state. fNIRS measured oxyhemoglobin levels in M1, premotor, and somatosensory areas during a 5-min resting-state recording (22, 23). RMT was also collected (Figure 1: CONSORT flow chart).

### 2.5 Statistical analysis

The FMA score was the primary outcome measure. Based on the literature (24), the mean FMA-UE scores in the sham group was reported as 11.1 ± 3.8. This value served as a reference for sample size estimation. It is expected that the score in the iTBS treatment group will improve by 3.8 points, with *α* = 0.05 and 90% power, the sample size was calculated using the following formula, *n* = 2(zα + zβ)^2^ * σ^2^ / δ^2^, 25 subjects per group were required, totaling 50 subjects after accounting for a 10% dropout rate. The database was managed and statistical analyses were conducted by an expert who was blinded to group allocation. Data processing was performed using SPSS 26.0. Normality was assessed with the Shapiro–Wilk test. Normal data were expressed as mean (±standard deviation, SD), non-normal data were expressed as median (interquartile range), and categorical variables as percentage (%). Within-group comparisons before and after treatment were conducted using the Wilcoxon signed-rank test, and between-group differences were assessed using the Mann–Whitney U test. Count data were analyzed with the chi-square test, and ordinal data comparisons were made using the Wilcoxon signed-rank test or Mann–Whitney U test.

fNIRS data were processed offline using MATLAB 2021 and Homer2 software. Motion artifacts were identified and corrected using wavelet filtering and spline interpolation, as implemented in the HOMER2 toolbox. Raw data were converted to optical density, corrected for motion artifacts, band-pass filtered, and converted to hemoglobin concentration changes. Six regions of interest (ROIs) related to motor processing were defined, including the M1, premotor cortex, and somatosensory areas. Functional connectivity was assessed using the Pearson correlation coefficient for ROI pairs (25). For ease of description, data from patients with right hemisphere damage were flipped. HbO2 amplitude and functional connectivity matrices at T1 and T2 were compared between iTBS and sham groups. For multiple comparisons the false discovery rate (FDR) method was used when analyzing functional connectivity matrices, with statistical significance set at *p* < 0.05. Pearson’s correlation coefficients (two-tailed) were used for the correlation analysis between changes in functional connectivity and clinical outcomes.

## 3 Results

### 3.1 Baseline characteristics of the patients

266 stroke inpatients were screened for eligibility. Of these, 50 patients met the inclusion criteria and were randomized into the iTBS or sham group. One patient in the sham-iTBS group refused to continue receiving the intervention due to early discharge, and another discontinued due to perceived ineffectiveness of the treatment. There were no significant differences in demographic and clinical data, NIHSS, modified Ashworth scale at baseline (Supplementary Table 1). Of the 50 patients, 30 participated in fNIRS measurements, with 15 patients completing the measurements in each group.

### 3.2 Safety outcome

Both the iTBS and the control group demonstrated excellent safety profiles, with no serious adverse reactions reported, including extreme events such as death or epilepsy. The occurrence of a local pain or discomfort in the stimulation area was more frequent in the iTBS group (10/250 sessions) compared to the sham group (3/230 sessions), although this difference did not attain statistical significance (*p* = 0.057). However, after a brief rest and re-adaptation period, they were both able to tolerate and accept the prescribed stimulation intensity. This discomfort may be related to inadequate preparation or expectations of the subjects. Overall, these data provide evidence that iTBS intervention is safe and reliable, with no serious adverse reactions observed, and the subjects demonstrated a high level of acceptance toward the iTBS intervention.

### 3.3 Primary outcomes

#### 3.3.1 FMA-UE

**Figure 2:**
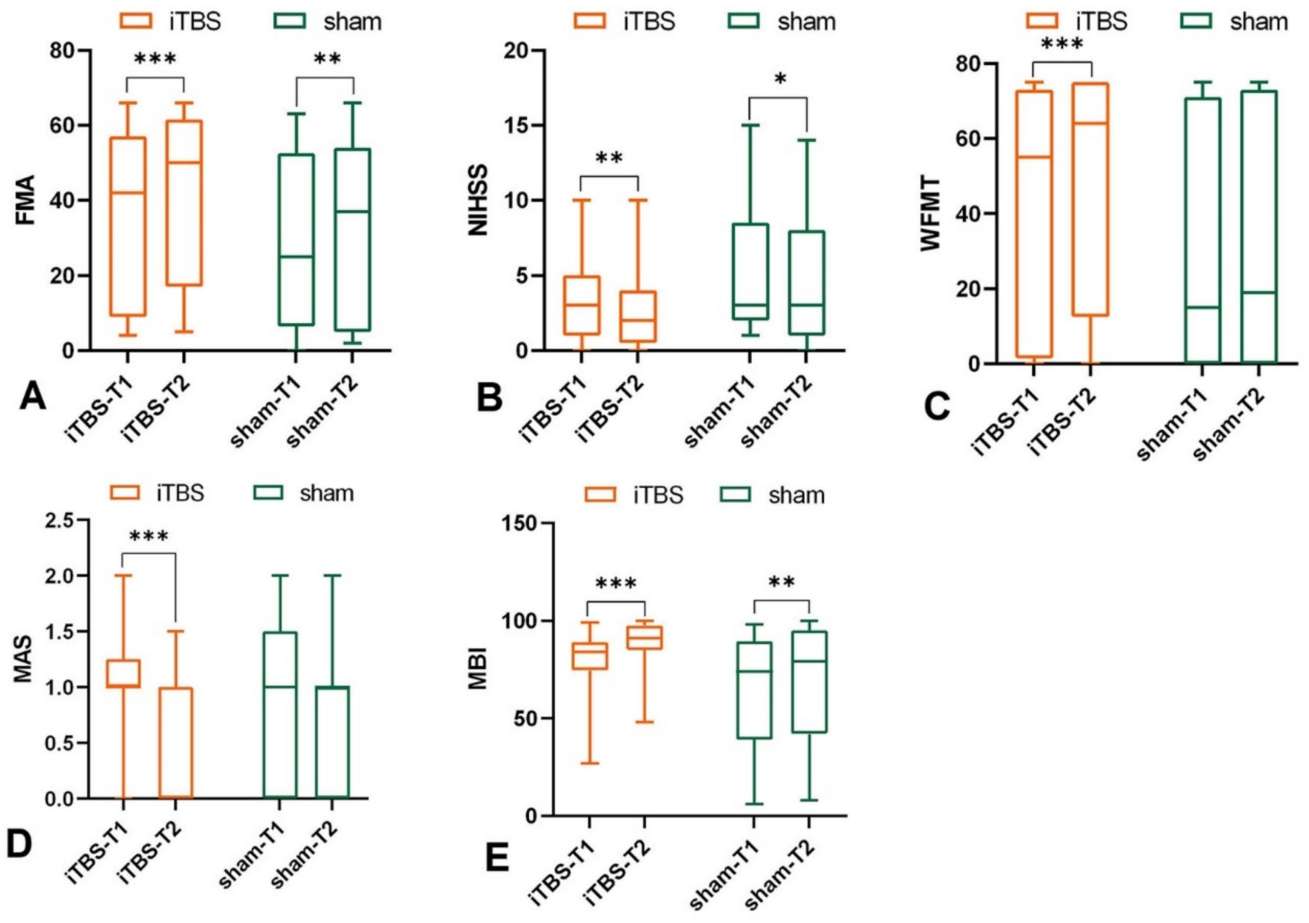
Effects of iTBS and sham stimulation on **(A)** FMA, **(B)** NIHSS, **(C)** WMFT, **(D)** MAS, and **(E)** MBI scores. T1 and T2 indicate measurements before and after intervention, respectively; *p < 0.05, **p < 0.01, ***p <0.001.

**Figure 3:**
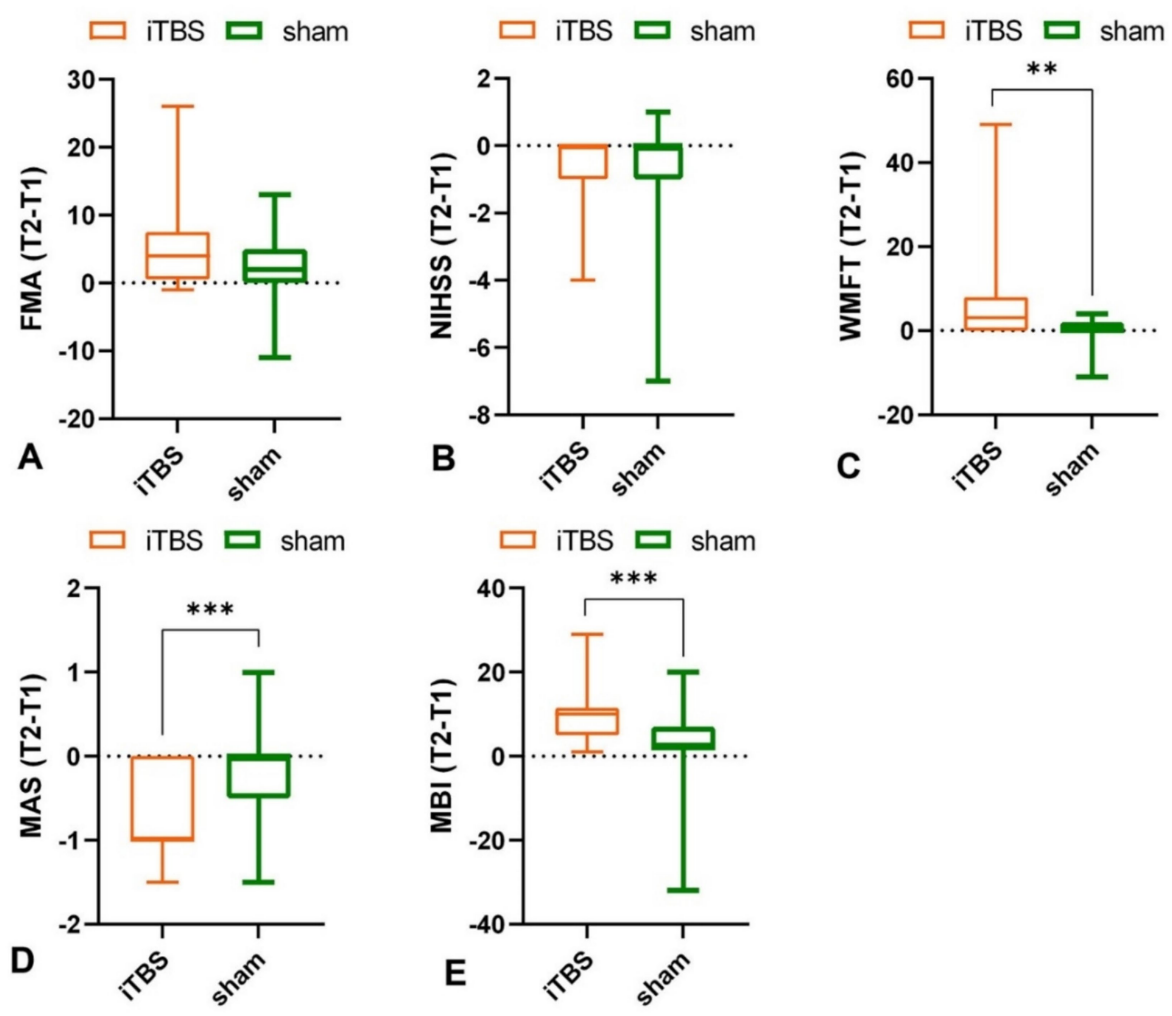
Effects of iTBS and sham stimulation on change of **(A)** FMA, **(B)** NIHSS, **(C)** WMFT, **(D)** MAS, and **(E)** MBI scores. *p < 0.05, **p < 0.01, ***p < 0.001.

Intra-group results: Both groups showed significant improvement after the intervention in FMA (iTBS group: *p* < 0.001; sham group: *p* = 0.005) (Figure 2A; Table 1). Inter-group results: No significant differences in the change scores for FMA-UE between groups after 10 sessions (*p* = 0.224, Cohen’s *d* = 0.39) (Figure 3A; Table 1). Although the median improvement for the iTBS group was numerically greater than that of the sham group (4.00 v. 2.00, respectively), the difference was not statistically significant. An analysis of covariance (ANCOVA) was conducted with post-treatment FMA-UE scores as the dependent variable, treatment group as the fixed factor, and baseline FMA-UE and time since onset as covariates. Baseline FMA-UE was a strong predictor of post-treatment outcomes (*F*(1,44) = 732.66, *p* < 0.001), while time since onset did not significantly influence the results (*F*(1,44) = 0.73, *p* = 0.398). After adjusting for these covariates, no significant group effect was observed (*F*(1,44) = 2.03, *p* = 0.161). Estimated marginal means indicated that the adjusted post-treatment FMA-UE score was 38.3 (SE = 1.10; 95% CI [36.1, 40.5]) in the iTBS group and 36.0 (SE = 1.15; 95% CI [33.7, 38.3]) in the sham group. Although the iTBS group showed a slightly higher adjusted mean score, this difference did not reach statistical significance. Although no statistically significant difference was observed in baseline FMA-UE scores between the groups, the iTBS group tended to have higher scores than the sham group.

**Table 1:** Inferential statistics of outcome measures.

Outcome	Intra-group				Inter-group	
	iTBS		Sham		iTBS	Sham
	*t*/*z*-value	*p* value	*t*/*z*-value	*p* value	*t*/*z*-value	*p* value
FMA	−3.854	<0.001***	−2.793	0.005**	−1.216	0.224
NIHSS	−3.133	0.002**	−2.068	0.039*	−0.869	0.385
WFMT	−3.728	<0.001***	−0.625	0.532	−3.074	0.002**
MAS	−3.900	<0.001***	−1.300	0.194	−3.200	0.001**
MBI	−4.378	<0.001***	−3.115	0.002**	−3.575	<0.001***
RMT	2.604	0.016*	0.894	0.381	0.900	0.373

*p < 0.05; **p < 0.01; ***p < 0.001.

### 3.4 Secondary outcomes

#### 3.4.1 NIHSS

Intra-group results: Both the iTBS group (*p* = 0.002), and sham group (*p* = 0.039) showed significant reductions in NIHSS total scores after 10 sessions compared to baseline (Figure 2B; Table 1). Inter-group results: There was no significant difference between groups in the change scores for NIHSS (*p* = 0.385, Cohen’s *d* = 0.01) (Figure 3B; Table 1).

#### 3.4.2 WMFT

Intra-group results: The iTBS group showed a significant improvement in WMFT scores compared to baseline (*p* < 0.001), whereas the sham group did not show a statistically significant difference (*p* = 0.532) (Figure 2C; Table 1). Inter-group results: After 10 sessions of treatment, the change scores of WMFT were significantly different between the two groups (*p* = 0.002, Cohen’s *d* = 0.78) (Figure 3C; Table 1). This indicates a statistically significant and moderate-to-large effect favoring the iTBS group, suggesting greater improvement in upper extremity motor performance in functional tasks compared to the sham group.

#### 3.4.3 MAS

Intra-group results: The iTBS group showed a significant improvement in muscle tone of wrist flexors compared to baseline (*p* < 0.001) (Figure 2D; Table 1), In contrast, the sham group did not show any significant change (*p* = 0.194, Table 1). Inter-group results: The iTBS group had a significantly greater reduction in MAS scores compared to the sham group (*p* = 0.001, Cohen’s *d* = 1.02) (Figure 3D; Table 1), indicating a large effect size favoring iTBS in reducing spasticity of the wrist flexors.

#### 3.4.4 MBI

Intra-group results: Both groups showed significant improvements in MBI scores post-treatment compared to baseline (iTBS group: *p* < 0.001; sham group: *p* = 0.002) (Figure 2E; Table 1). Inter-group results: The iTBS group demonstrated a significantly greater improvement in MBI scores compared to the sham group (*p* < 0.001, Cohen’s *d* = 0.88) (Figure 3E; Table 1), indicating a large effect size. iTBS yielded a greater improvement in the quality of life than sham did.

### 3.5 Cortical excitability

#### 3.5.1 RMT

**Figure 4:**
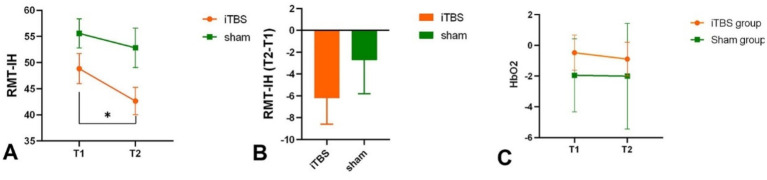
RMT in intact hemisphere intra-group **(A)** and inter-group **(B)**. HbO2 in each group in T1 and T2 **(C)**. *p < 0.05.

Intra-group results: The iTBS group showed a significant decrease in RMT at T2 compared to T1 (*p* = 0.016) (Figure 4A; Table 1). Inter-group results: There was no significant difference in the change scores of RMT on the unaffected side between the iTBS and sham groups (*p* = 0.373, Cohen’s *d* = −0.26) (Figure 4B; Table 1).

#### 3.5.2 fNIRS

**Figure 5:**
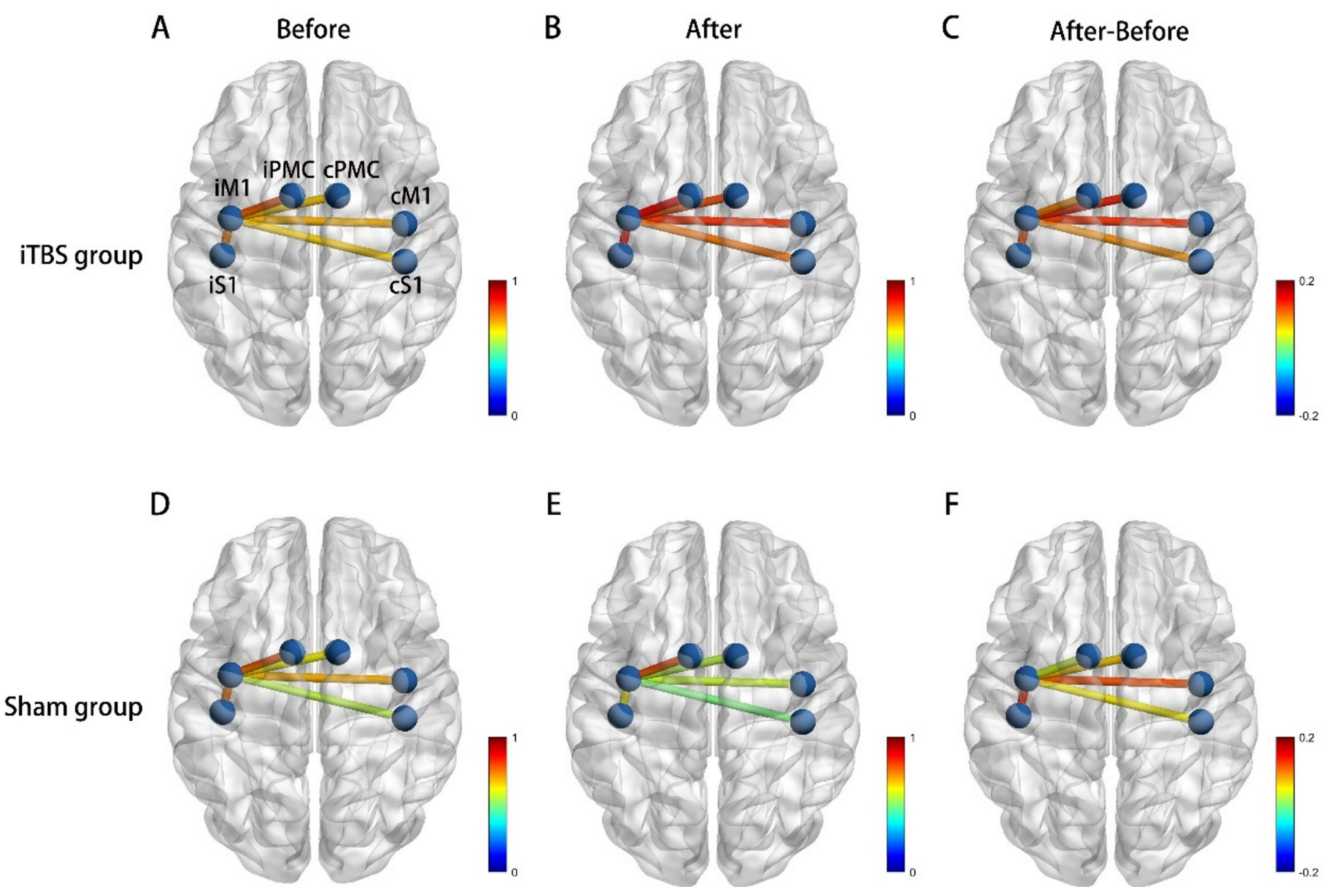
Functional connectivity (correlation) change, **(A)** Correlations between each ROI at baseline in iTBS group. **(B)** Correlations between each ROI after iTBS. **(C)** Correlations between iM1 cortex and cM1 was significantly increased after iTBS. **(D)** Correlations between each ROI at baseline in sham group. **(E)** Correlations between each ROI after sham stimulation. **(F)** The change of correlation between each ROI after sham stimulation. iM1, Ipsilesional Motor Cortex, cM1, contralesional Motor Cortex, iPMC, ipsilesional Premotor Cortex, cPMC, contralesional Premotor Cortex, iS1, ipsilesional Somatosensory Cortex, cS1, contralesional Somatosensory Cortex.

Intra-group results: There was no significant difference in the average HbO2 amplitude of M1 between T1 and T2 in both the iTBS group (*p* = 0.846) and the sham group (*p* = 0.192) (Figure 4C). Inter-group results: No significant difference was found in the averaged HbO2 amplitude between the two groups before or after treatment (*p* = 0.29). However, increased connectivity was observed in the iTBS group: there was a significant correlation between iM1 and cM1 (*p* = 0.009, uncorrected; *p* = 0.049, FDR corrected), and between iM1 and cPMC (*p* = 0.029, uncorrected; *p* = 0.147, FDR corrected) compared to the sham group (Figure 5). A correlation analysis was performed to assess the association between changes in functional connectivity (FC_change) and clinical outcomes following iTBS intervention. A significant moderate correlation was observed between FC_change and WMFT_change (*r* = 0.585, *p* = 0.022), indicating that an increase in FC was associated with a greater change in WMFT scores.

## 4 Discussion

### 4.1 Neurological deficit

The study demonstrated that both the combination of iTBS and conventional neurorehabilitation therapy and the traditional therapy alone led to improved neurological outcomes in subacute stroke patients. Hsu et al. found significant improvements in NIHSS scores with real iTBS compared to sham stimulation over 10 days (26), while our study did not show significant differences in NIHSS changes between groups. The results were comparable to those of Hosomi et al., who conducted a series of 10 daily 5-Hz rTMS of the ipsilesional primary motor cortex and observed an improvement in NIHSS scores from the baseline, though not to a level that differed significantly from the sham group (27). Other research highlights that early neurorehabilitation is most effective within the first three months post-stroke (28). Our study found that patients in both groups had fewer neurological deficits after treatment compared to baseline in patients with subacute ischemic stroke.

### 4.2 Motor function

The FMA showed significant improvement in both the iTBS group and the Sham group. While the median improvement observed in the iTBS group exceeded that of the sham group numerically, the disparity failed to reach statistical significance. This aligns with Watanabe et al., who observed a significant increase in the overall FMA score, although the differences did not reach statistical significance in the acute phase following iTBS stimulation (12). The FMA may exhibit a ceiling effect in mildly to moderately impaired patients, which may have limited our ability to detect between-group differences. In contrast, Zhang et al. identified a significant time effect and time-by-group interaction effect in chronic stroke patients, although the advantage diminished at follow-up (29). Specifically, WMFT scores improved significantly in the iTBS group, with a substantial difference between the iTBS and sham groups. The WMFT, focused on functional tasks and motor performance, is more sensitive to detecting subtle improvements in activities of daily living compared to the FMA. The iTBS intervention demonstrated a significant improvement in the WMFT scores, suggesting a positive impact and a more pronounced effect on the ability to perform functional tasks. This may be attributed to the focal modulation of the hand area in the iM1, where iTBS was targeted. Previous studies suggest that fine motor recovery is more sensitive to changes in cortical excitability and neuroplasticity, which may be selectively promoted by iTBS through long-term potentiation-like effects. Similarly, Kakuda et al. applied rTMS to contralateral cerebral cortex and observed a notable reduction in the time required to complete the WMFT (30).

### 4.3 Muscle tone

MAS scores significantly improved in the iTBS group both within the group and when compared to the sham group. Specifically, the MAS scores showed a statistically significant difference post-treatment within the iTBS group, and the difference in MAS scores between the iTBS and sham groups was also statistically significant. Our findings are consistent with the finding from Barros Galvão et al. indicating that rTMS showed a significant reduction of spasticity in patients with chronic stroke (11, 31, 32). Our current study supports previous meta-analytic conclusions, further confirming that iTBS effectively reduces muscle spasticity in subacute ischemic stroke patients (10). The reduction in spasticity likely resulted from the modulation of cortical excitability, which promotes neural plasticity and functional recovery. This supports the notion that iTBS can be an effective intervention for reducing spasticity in the subacute phase of stroke.

### 4.4 ADL

The MBI scores showed significant improvements within both the iTBS and sham groups when comparing post-intervention scores to pre-intervention scores. Furthermore, the change in MBI scores was significantly greater in the iTBS group than in the sham group. These findings suggest that both active and sham treatments contributed to improvements in activities of daily living, potentially resulted from the effects of conventional neurorehabilitation provided during the intervention (33). However, the greater improvement in the iTBS group supports the notion that iTBS has a more substantial impact on functional recovery and quality of life in stroke patients, reinforcing its potential as an effective therapeutic intervention.

### 4.5 Cortical excitability

The RMT in cM1 was found to be significantly lower in iTBS group when comparing post-intervention to pre-intervention, while no significant change was observed in the sham group. This finding is consistent with the known effects of iTBS, which is thought to increase cortical excitability. The theoretical rationale for targeting the ipsilesional motor cortex with facilitatory iTBS is based on the evidence that an increase in the cortical excitability of affected hemisphere correlates with improved motor function in the paretic paretic upper limb (34). The decrease in motor threshold following iTBS may be attributed to cortical excitability, likely through mechanisms such as increased neuroplasticity and functional connectivity. It was found that the IH may play a pivotal role in recovery after stroke, as indicated by the deterioration of functional performance after cTBS (35). The decreased RMT reflects enhanced corticospinal excitability, which may facilitate motor relearning and voluntary movement execution, contributing to improved function. Interestingly, although there were no significant changes in HbO2 levels in either group, the functional connectivity analysis revealed a significant enhancement between the iM1 and the cM1 in the iTBS group post-intervention. This finding is not unexpected, as iTBS is primarily aimed at modulating cortical excitability rather than affecting global physiological parameters like oxygen saturation. In line with previous studies (25, 36), we found that iTBS did not alter HbO2 concentration. It may promote functional recovery or enhancement through other mechanisms, such as improving brain functional connectivity. The significant enhancement in functional connectivity between the iM1 and cM1 regions following iTBS intervention indicates that the treatment may have facilitated interhemispheric communication. It remains controversial whether applying depressive stimulation to the contralesional hemisphere to reduce abnormal transcallosal inhibition can effectively improve movement of the affected limb, particularly in the early phase of stroke recovery. This is because interhemispheric inhibition may not have fully developed within the first three months after stroke onset (37). For example, recruitment of the cM1 appears to be necessary for recovery in patients with severe UE impairment during the subacute phase of ischemic stroke (3). The study found that after receiving cTBS to the cM1, patients experienced a temporary worsening of upper limb function. This suggests that excessive inhibition of the cM1 region may be detrimental to the recovery process following a stroke. Also, Nicolo et al. observed that an inhibition of cM1 or a reduction of interhemispheric interactions did not lead to improved motor recovery (38). Taken together, these findings suggest that the aim to balance the interaction between hemispheres may be oversimplified. Overall, our findings reinforce the therapeutic potential of iTBS in promoting recovery through the modulation of cortical excitability and enhancement of functional connectivity.

## 5 Conclusion

In summary, our study demonstrates that while iTBS combined with standard rehabilitation protocols did not yield statistically significant improvements in the primary outcome compared to sham treatment, it was associated with notable improvements in secondary outcomes, including muscle tone, motor performance, and functional connectivity in subacute ischemic stroke patients. These findings suggest that iTBS may offer added benefits for specific functional measures and quality of life, highlighting its potential as a supplementary approach in post-stroke rehabilitation.

### 5.1 Limitations

Our study has several limitations. It may be noted that, although the adjusted analysis did not reveal a statistically significant group effect, the estimated marginal means suggested a trend toward higher FMA-UE scores in the iTBS group. This finding, together with prior reports, indicates that iTBS may provide additional benefits, which should be verified in larger, adequately powered studies. These imbalances, although statistically non-significant, may have influenced the observed effects and should be taken into account when interpreting the findings. The small sample size may limit generalizability, and longer follow-up is needed to assess the durability of improvements. Additional outcome measures could provide a more comprehensive evaluation. Blinding may not have been perfect, potentially introducing bias, and the study’s focus on brain infarction may limit applicability to other stroke types or neurological disorders. The relatively small fNIRS subsample could limit statistical power and increase the risk of Type II errors. Future research should address these issues to confirm and expand upon our findings.

## Data Availability

The raw data supporting the conclusions of this article will be made available by the authors, without undue reservation.
