# Supplementary material for: The effectiveness of intermittent theta burst stimulation for upper limb motor recovery after stroke: an exploratory randomized controlled trial
Source: Front Neurol. 2025 Sep 12;16:1634277. doi: 10.3389/fneur.2025.1634277 (PMC12464961; doi:10.3389/fneur.2025.1634277)
Supplement: Supplementary file 1 [file Table_1.docx]

Table1.Characteristics of the participants.

| Variable | iTBS group  (n= 25) | Sham group  (n= 23) | *p*-value |
| --- | --- | --- | --- |
| Age (y), mean ± SD | 66.48±11.72 | 67.65±12.34 | 0.737 |
| Gender: male, n (%) | 16 (64%) | 12 (52.2%) | 0.406 |
| Time since the stroke (m) | 1.00 (1.00) | 2.00 (2.00) | 0.310 |
| Paretic side: left, n (%) | 16 (64%) | 10 (43.5) | 0.154 |
| NIHSS score | 3.36±2.51 | 4.96±4.10 | 0.117 |
| Initial FMA-UE scores | 42.00 (48.00) | 25.00 (48.00) | 0.215 |
| Initial MAS scores | 1.00(0.30) | 1.00(1.50) | 0.645 |
| Initial MBI scores | 84.00(15.00) | 74.00 (51.00) | 0.179 |
| Initial RMT, % | 48.84±14.41 | 55.57±13.28 | 0.100 |

Values presented are mean ± SEM or median (IQR). NIHSS, National Institutes of Health Stroke Scale; FMA-UE, Fugl-Meyer Assessment for the Upper Extremity; MAS, Modified Ashworth Scale; MBI, modified Barthel Index, RMT, rest motor threshold.

Table2. Descriptive of outcome measures

| Outcome | Pre-treatment | | Post-treatment | | Mean change score | |
| --- | --- | --- | --- | --- | --- | --- |
|  | iTBS | Sham | iTBS | Sham | iTBS | Sham |
| FMA | 42.00(48.00) | 25.00(48.00) | 50.00(45.00) | 37.00(49.00) | 4.00(7.00) | 2.00(5.00) |
| NIHSS | 3.00(4.00) | 3.00(6.00) | 2.00(4.00) | 3.00(7.00) | 0.00(1.00) | 0.00(1.00) |
| WMFT | 5.50(72.00) | 15.00(71.00) | 64.00(63.00) | 19.00(73.00) | 3.00(8.00) | 0.00(2.00) |
| MAS | 1.00(0.30) | 1.00(1.50) | 0.00(1.00) | 1.00(1.00) | -1.00(1.00) | 0.00(0.50) |
| MBI | 84.00(15.00) | 74.00(51.00) | 91.00(13.00) | 79.00(53.00) | 10.00(7.00) | 2.00(5.00) |
| RMT | 48.84±14.41 | 55.57±13.28 | 42.64±2.62 | 52.83±3.79 | -6.20±2.38 | -2.74±3.07 |

Values presented are mean ± SEM or median (IQR).
